# Efficient Quantum Private Comparison Based on GHZ States

**DOI:** 10.3390/e26050413

**Published:** 2024-05-10

**Authors:** Min Hou, Yue Wu, Shibin Zhang

**Affiliations:** 1 School of Computer Science, Sichuan University Jinjiang College, Meishan 620860, China; houmin@scujj.edu.cn (M.H.); ywu@uestc.edu.cn (Y.W.); 2 Network and Data Security Key Laboratory of Sichuan Province, University of Electronic Science and Technology of China, Chengdu 610054, China; 3 School of Cybersecurity, Chengdu University of Information Technology, Chengdu 610225, China; 4 Advanced Cryptography and System Security Key Laboratory of Sichuan Province, Chendu 610225, China

**Keywords:** quantum private comparison (QPC), rotation operation, GHZ states, efficiency

## Abstract

Quantum private comparison (QPC) is a fundamental cryptographic protocol that allows two parties to compare the equality of their private inputs without revealing any information about those inputs to each other. In recent years, QPC protocols utilizing various quantum resources have been proposed. However, these QPC protocols have lower utilization of quantum resources and qubit efficiency. To address this issue, we propose an efficient QPC protocol based on GHZ states, which leverages the unique properties of GHZ states and rotation operations to achieve secure and efficient private comparison. The secret information is encoded in the rotation angles of rotation operations performed on the received quantum sequence transmitted along the circular mode. This results in the multiplexing of quantum resources and enhances the utilization of quantum resources. Our protocol does not require quantum key distribution (QKD) for sharing a secret key to ensure the security of the inputs, resulting in no consumption of quantum resources for key sharing. One GHZ state can be compared to three bits of classical information in each comparison, leading to qubit efficiency reaching 100%. Compared with the existing QPC protocol, our protocol does not require quantum resources for sharing a secret key. It also demonstrates enhanced performance in qubit efficiency and the utilization of quantum resources.

## 1. Introduction

Quantum private comparison (QPC) plays a crucial role in secure multi-party computation and privacy-preserving applications. It enables two parties, Alice and Bob, to compare their private inputs without disclosing any information about those inputs to each other or to any eavesdroppers. Traditional classical private comparison protocols are inherently vulnerable to information leakage because they rely on the assumption of number-theoretical complexity, which is no longer reliable due to the emergence of quantum algorithms (e.g., Shor’s algorithm [1] and Grover’s algorithm [2]). QPC, on the other hand, leverages the unique properties of quantum mechanics (such as quantum entanglement, non-cloning, the uncertainty principle, and the superposition principle) to conduct secure comparisons while safeguarding the privacy of the inputs and ensuring information-theoretic security.

The first QPC protocol was suggested by Yang and Wen [3], utilizing two-photon entangled states and four unitary operations to achieve the comparison. Decoy photons and hash functions are used to prevent eavesdropping on players’ private inputs. In 2010, triplet GHZ states and single-particle measurements were used to develop an efficient QPC protocol [4]. This protocol divides secret messages into multiple groups, resulting in saved quantum resources. Nevertheless, Ref. [4] was susceptible to quantum attacks and, thus, results in information leakage. Some improvements have been proposed to enhance its security [5]. Then, Tseng et al. [6] utilized quantum entanglement of Bell states to propose an easier implementation of the QPC protocol, achieving a qubit efficiency of 50%. Jia et al. [7] employed the entanglement properties of χ-type states as information carriers to accomplish private comparison. Local unitary operations are used to encode private information, and joint measurements are adopted to extract the results. Quantum superdense coding is utilized to achieve higher efficiency. Since then, several QPC protocols have been proposed, utilizing various quantum resources such as single photons [8,9,10,11], entangled states [12,13,14,15,16,17,18,19,20,21], and cluster states [22,23,24,25,26]. Additionally, two-atom product states and single-atom measurements are used in a QPC protocol [27]. This protocol enables the comparison of the equality of one classical bit in each round, resulting in the qubit efficiency reaching 50%. Another QPC protocol, which does not require any classical computation, was proposed by Lang [28] in 2020. In this protocol, quantum gates are utilized for classical calculations instead of the bitwise XOR operation, leading to improved security. Zhang et al. [29] utilized quantum homomorphic encryption to develop a multi-party QPC protocol with a TP who will faithfully perform homomorphic calculations, effectively reducing the quantum resources required. A different QPC protocol, developed by Huang et al. in 2023 [30], is used to determine the equality of single-qubit states. This protocol utilizes more accessible quantum technologies, such as rotation encryption and swap test, to compare qubits.

According to the analysis of previous QPC protocols, while the aforementioned QPC protocols have shown potential for achieving secure private comparison, they often encounter challenges related to the utilization of quantum resources and qubit efficiency. For example, in most cases, the equality of one classical bit can be compared with Bell states and GHZ states. The qubit efficiency only reaches 50% and 33%, respectively, which limits qubit efficiency. In addition, most QPC protocols require the implementation of QKD protocol to share a secret key used for encrypting private inputs, resulting in a decrease in the utilization of quantum states. Therefore, appropriate measures need to be selected to enhance the qubit efficiency of QPC protocols. Rotation operations, which are special unitary operations, have received widespread attention. Various rotation-operation-based quantum secure protocols have been proposed, such as quantum secret sharing (QSS) [31], quantum signature [32], and quantum key agreement [33]. Rotation operations can also be applied in designing QPC protocols due to their unique properties.

In this paper, we utilize GHZ states and rotation operations to propose an efficient QPC protocol, which achieves a qubit efficiency of 100% and a higher utilization rate of quantum resources. In our protocol, two users encode their secrets into the received quantum sequence, that is, performing the corresponding rotation operation on the received GHZ states. The secrets can be privately compared with a TP who will not deviate from the protocol execution or conspire with any participant, but may attempt to obtain the inputs of the users by learning the immediate data. The function of the TP is to prepare and encrypt an initial quantum sequence contained in GHZ states at the beginning and, then, decrypt and measure the decrypted quantum sequence at the end.

The main contributions of our paper are as follows.

(1) Our protocol does not require QKD protocol for sharing a secret key to ensure the security of the inputs. This results in no consumption of quantum resources for key sharing.
(2) The quantum sequence is transmitted between the TP and two users in a circular mode. The inputs of the two users are encoded into the transmitted quantum sequence, leading to the multiplexing of quantum resources and improving the utilization of quantum resources.
(3) One GHZ state can be compared to three-bit classical information, enabling qubit efficiency to reach 100%.

The rest of this paper is structured as follows. Preliminary knowledge is introduced in Section 2. The proposed quantum private comparison based on GHZ states is presented in Section 3. A simulation experiment demonstrating the correctness and feasibility of our protocol is outlined in Section 4. Security analysis and qubit efficiency are discussed in Section 5 and Section 6, respectively. Finally, the conclusion is provided in Section 5.

## 2. Preliminary Knowledge

Eight types of GHZ states in our protocol are denoted as

(1) $$|\varphi_{1}\rangle =\frac{1}{\sqrt{2}}\left(|000\rangle +|111\rangle \right)$$

(2) $$|\varphi_{2}\rangle =\frac{1}{\sqrt{2}}\left(|000\rangle -|111\rangle \right)$$

(3) $$|\varphi_{3}\rangle =\frac{1}{\sqrt{2}}\left(|100\rangle +|011\rangle \right)$$

(4) $$|\varphi_{4}\rangle =\frac{1}{\sqrt{2}}\left(|100\rangle -|011\rangle \right)$$

(5) $$|\varphi_{5}\rangle =\frac{1}{\sqrt{2}}\left(|010\rangle +|101\rangle \right)$$

(6) $$|\varphi_{6}\rangle =\frac{1}{\sqrt{2}}\left(|010\rangle -|101\rangle \right)$$

(7) $$|\varphi_{7}\rangle =\frac{1}{\sqrt{2}}\left(|110\rangle +|001\rangle \right)$$

(8) $$|\varphi_{8}\rangle =\frac{1}{\sqrt{2}}\left(|110\rangle -|001\rangle \right)$$

The rotation operation is denoted as

(9) $$R_{y}\left(\theta \right)=\left(\begin{array}{cc} \cos\frac{\theta }{2} & -\sin\frac{\theta }{2}\\ \sin\frac{\theta }{2} & \cos\frac{\theta }{2} \end{array}\right)$$

Equation (9) represents a unitary matrix since $R_{y}^{†}\left(\theta \right)R_{y}\left(\theta \right)=I$, and it can be implemented by rotating around the y-axis with θ on the Bloch sphere.

When performing rotation operations on GHZ states, we observe the following two features.

**Lemma 1.**  *For any $\left|\varphi_{i}\right.\rangle \left(i=1,2,\cdots ,8\right)$, $\left(R_{y}\left(-\theta_{1}\right)⊗R_{y}\left(-\theta_{2}\right)⊗R_{y}\left(-\theta_{3}\right)\right)\left(R_{y}\left(\theta_{1}\right)⊗R_{y}\left(\theta_{2}\right)⊗R_{y}\left(\theta_{3}\right)\right)\left|\varphi_{i}\right.\rangle =\left|\varphi_{i}\right.\rangle$ holds.*

**Proof.**  Let us consider $\left|\varphi_{1}\rangle \right.$ as an example. We have the following equation.

(10) $$\begin{array}{l} \left(R_{y}\left(-\theta_{1}\right)⊗R_{y}\left(-\theta_{2}\right)⊗R_{y}\left(-\theta_{3}\right)\right)\left(R_{y}\left(\theta_{1}\right)⊗R_{y}\left(\theta_{2}\right)⊗R_{y}\left(\theta_{3}\right)\right)|\varphi_{1}\rangle \\ =\frac{1}{\sqrt{2}}\left(R_{y}\left(-\theta_{1}\right)⊗R_{y}\left(-\theta_{2}\right)⊗R_{y}\left(-\theta_{3}\right)\right)\left(\begin{array}{l} R_{y}\left(\theta_{1}\right)|0\rangle ⊗R_{y}\left(\theta_{2}\right)|0\rangle ⊗R_{y}\left(\theta_{3}\right)|0\rangle \\ +R_{y}\left(\theta_{1}\right)|1\rangle ⊗R_{y}\left(\theta_{2}\right)|1\rangle ⊗R_{y}\left(\theta_{3}\right)|1\rangle \end{array}\right)\\ =\frac{1}{\sqrt{2}}\left(\begin{array}{l} R_{y}\left(0\right)|0\rangle ⊗R_{y}\left(0\right)|0\rangle ⊗R_{y}\left(0\right)|0\rangle \\ +R_{y}\left(0\right)|1\rangle ⊗R_{y}\left(0\right)|1\rangle ⊗R_{y}\left(0\right)|1\rangle \end{array}\right)\\ =\frac{1}{\sqrt{2}}\left(|0\rangle ⊗|0\rangle ⊗|0\rangle +|1\rangle ⊗|1\rangle ⊗|1\rangle \right)\\ =|\varphi_{1}\rangle \end{array}$$

In the same way, we can prove that

(11) $$\left(R_{y}\left(-\theta_{1}\right)⊗R_{y}\left(-\theta_{2}\right)⊗R_{y}\left(-\theta_{3}\right)\right)\left(R_{y}\left(\theta_{1}\right)⊗R_{y}\left(\theta_{2}\right)⊗R_{y}\left(\theta_{3}\right)\right)|\varphi_{i}\rangle =|\varphi_{i}\rangle$$

Thus, Lemma 1 holds. □

**Lemma 2.**  *For $\theta_{j}\in \left\{0,\pi \right\},j\in \left\{1,2,3\right\}$,* *the resultant states, without considering the global phase, are shown in Table 1 when performing the corresponding rotation operation $R_{y}\left(\theta_{j}\right)$ on each particle of different GHZ states.*

## 3. Quantum Private Comparison Based on GHZ States

In this section, we will provide detailed steps of the proposed QPC protocol, where a semi-honest TP assists two distrustful players, Alice and Bob, in comparing whether their secrets are equal. The semi-honest TP will not deviate from the protocol execution or conspire with any participant, but may try to obtain useful information about the users’ inputs through illicit means. 

**Figure 1:**
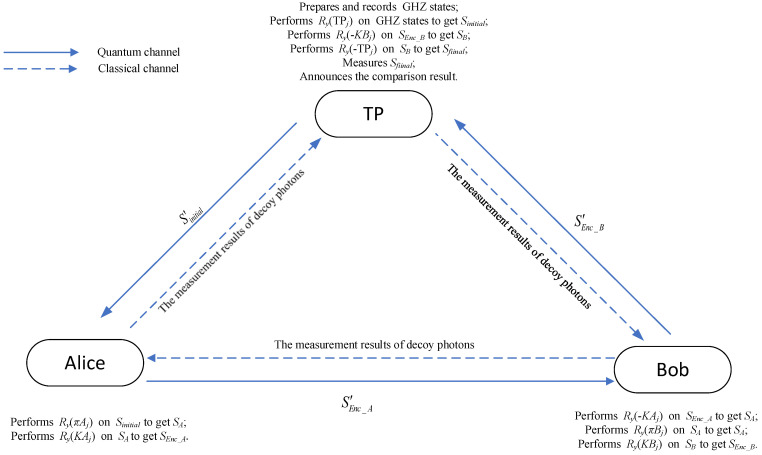
The diagram of the QPC protocol.

Suppose that Alice and Bob hold their private integers denoted as $I_{a}$ and $I_{b}$, respectively. $I_{a}$ and $I_{b}$ can be represented in binary form as $X=\left\{x_{1},x_{2},\cdots ,x_{N}\right\}$, $Y=\left\{y_{1},y_{2},\cdots ,y_{N}\right\}$, respectively, where $x_{i},y_{i}\in \left\{0,1\right\}$,$\;i=\left\{1,2,\cdots ,N\right\}$, *N* is the length of the secrets, $X=\sum_{i=1}^{N}x_{i}2^{i-1}$, and $Y=\sum_{i=1}^{N}y_{i}2^{i-1}$. We assume that the quantum channel in the communication process is noiseless and lossless, while the classical channel is authenticated. By authenticating the classical channel, the identities of all communication parties can be verified, ensuring that only legitimate entities participate in the execution of the protocol. The detailed steps of the proposed QPC protocol based on GHZ states are as follows, and its diagram is depicted in Figure 1.

**Step 1.** Alice and Bob divide an *N*-bit string *X* and *Y* into N/3 groups, respectively. Each group consists of 3-bit classical information. If Nmod3≠0, fill in $3-\left(N\mathrm{mod}3\right)$ 0 for the last group. The *N*-bit strings *X* and *Y* are converted to $X=\left(A_{1},A_{2},\cdots ,A_{N/3}\right)$ and $Y=\left(B_{1},B_{2},\cdots ,B_{N/3}\right)$, respectively, where $A_{j}=\left(a_{j}^{1},a_{j}^{2},a_{j}^{3}\right)$, $B_{j}=\left(b_{j}^{1},b_{j}^{2},b_{j}^{3}\right)$ and j=1,2,⋯,N/3. 

**Step 2.** TP prepares N/3 *GHZ* states selected from Equations (1)–(8) and records these states. Then, she generates a secret key $\Theta_{TP}=\left(TP_{1},TP_{2},\cdots ,TP_{N/3}\right)$, where $TP_{j}=\left(tp_{j}^{1},tp_{j}^{2},tp_{j}^{3}\right)\in [\left.0,2\pi \right)$ and j=1,2,⋯,N/3. Finally, she performs rotation operations $R_{y}\left(TP_{j}\right)$ on the *j*-th GHZ states to obtain a sequence $S_{initial}$.

**Step 3.** TP prepares 3*M* photons randomly chosen from four quantum states $\left|0\right.,\left|1\right.\rangle ,\left|+\right.\rangle$ and $\left|-\right.\rangle$ as decoy states. These photons are then inserted into $S_{initial}$ at random positions to obtain a new sequence $S_{initial}^{\prime }$. The corresponding states and positions of decoy states are recorded by TP who will then send the sequence $S_{initial}^{\prime }$ to Alice.

**Step 4.** Upon receiving the sequence $S_{initial}^{\prime }$, Alice sends an acknowledgment to TP who will verify the presence of eavesdroppers. TP announces the corresponding bases and positions of decoy states to Alice, who will then conduct measurements on these states and return the measurement outcomes to TP. TP detects the presence of eavesdroppers in the transmission channel by comparing the consistency of the measurement outcomes with the initially prepared decoy states and calculates the error rate. If the error rate exceeds a predefined threshold, this protocol will be rebooted. Otherwise, the protocol proceeds to the following steps.

**Step 5.** Alice discards decoy states in $S_{initial}^{\prime }$ to obtain $S_{initial}$ and performs rotation operations $R_{y}\left(\pi A_{j}\right)$ on the *j*-th states in $S_{initial}$ to obtain a sequence $S_{A}$. She then generates her secret key $\Theta_{KA}=\left(KA_{1},KA_{2},\cdots ,KA_{N/3}\right)$, where $KA_{j}=\left(ka_{j}^{1},ka_{j}^{2},ka_{j}^{3}\right)\in \left.[0,\;2\pi \right)$ and j=1,2,⋯,N/3, and performs rotation operations $R_{y}\left(KA_{j}\right)$ on the *j*-th states in $S_{A}$ to obtain a sequence $S_{Enc\_A}$. To prevent eavesdropping, Alice randomly selects 3*M* photons from four quantum states $\left|0\right.,\left|1\right.\rangle ,\left|+\right.\rangle$ and $\left|-\right.\rangle$ as decoy states. These photons are then inserted into $S_{Enc\_A}$ at random positions to obtain a new sequence $S_{Enc\_A}^{\prime }$. Alice records the corresponding states and positions of decoy states and sends the sequence $S_{Enc\_A}^{\prime }$ to Bob. 

**Step 6.** Upon receiving the sequence $S_{Enc\_A}^{\prime }$, Bob interacts with Alice to check for the presence of eavesdroppers in the transmission process, similar to Step 4. If no outsider eavesdropper exists, Alice announces her secret key $\Theta_{KA}$ to Bob, who will then discard decoy states in $S_{Enc\_A}^{\prime }$ to obtain $S_{Enc\_A}$ and perform rotation operations $R_{y}\left(-KA_{j}\right)$ on the *j*-th states in $S_{Enc\_A}$ to recover the sequence $S_{A}$. 

**Step 7.** Bob performs rotation operations $R_{y}\left(\pi B_{j}\right)$ on the j-th states in $S_{A}$ to obtain a sequence $S_{B}$. He then generates his secret key $\Theta_{KB}=\left(KB_{1},KB_{2},\cdots ,KB_{N/3}\right)$, where $KB_{j}=\left(kb_{j}^{1},kb_{j}^{2},kb_{j}^{3}\right)\in [\left.0,\;2\pi \right)$ and j=1,2,⋯,N/3, and performs rotation operations $R_{y}\left(KB_{j}\right)$ on the j-th states in $S_{B}$ to obtain a sequence $S_{Enc\_B}$. To thwart potential external attacks by eavesdroppers, Bob randomly inserts 3M photons into $S_{Enc\_B}$ at various positions to obtain a new sequence $S_{Enc\_B}^{\prime }$. These photons are chosen from four quantum states $\left|0\right.,\left|1\right.\rangle ,\left|+\right.\rangle$ and $\left|-\right.\rangle$ as decoy states. Bob records the states and positions of decoy states and sends the sequence $S_{Enc\_B}^{\prime }$ to TP. 

**Step 8.** Upon receiving the sequence $S_{Enc\_B}^{\prime }$, TP interacts with Bob to detect eavesdropping, similar to Step 4. If no eavesdropper exists in the transmission process, Bob will announce his secret key $\Theta_{KB}$ to TP, who will then discard the decoy states in $S_{Enc\_B}^{\prime }$ to obtain $S_{Enc\_B}$ and perform rotation operations $R_{y}\left(-KB_{j}\right)$ on the *j*-th states in $S_{Enc\_B}$ to recover the sequence $S_{B}$. 

**Step 9.** TP performs rotation operations $R_{y}\left(-TP_{j}\right)$ on the *j*-th GHZ states in $S_{B}$ to obtain a sequence $S_{final}$ and, then, conducts GHZ-basis measurements on the *j*-th states in $S_{final}$ to obtain the measurement outcomes. If each measurement outcome is consistent with the initially prepared GHZ states in Step 2, then X=Y. Otherwise, X≠Y. TP announces the comparison results to Alice and Bob.

## 4. Simulation Experiment

In this section, we utilize a concrete example and its simulation on IBM Quantum Composer to show the correctness and feasibility of our protocol. Suppose that the private integers of Alice and Bob are *X* = 10 and *Y* = 18, which can be represented in binary form as $X=\left(x_{1},x_{2},x_{3},x_{4}\right)=0101$ and $Y=\left(y_{1},y_{2},y_{3},y_{4},y_{5}\right)=01001$, respectively. Alice and Bob divide the strings *X* and *Y* into two groups, $X=\left(A_{1},A_{2}\right)=\left(x_{1}x_{2}x_{3},x_{4}\right)$ and $Y=\left(B_{1},B_{2}\right)=\left(y_{1}y_{2}y_{3},y_{4}y_{5}\right)$. Since the lengths of *X* and *Y* are not multiples of three, Alice and Bob will add two zero and one zero in the group of $A_{2}$ and $B_{2}$, respectively. Thereafter, $A_{1}=\left(x_{1}x_{2}x_{3}\right)=\left(010\right)$, $A_{2}=\left(x_{4}00\right)=\left(100\right)$, $B_{1}=\left(y_{1}y_{2}y_{3}\right)=\left(010\right)$, $B_{2}=\left(y_{4}y_{5}0\right)=\left(010\right)$. 

We assume that the semi-honest TP prepares two GHZ states denoted as $\left|\varphi_{1}\rangle \right.$ and $\left|\varphi_{6}\rangle \right.$ and the secret key $\Theta_{TP}=\left(TP_{1},TP_{2}\right)=\left(\left(tp_{1}^{1},tp_{1}^{2},tp_{1}^{3}\right),\;\left(tp_{2}^{1},tp_{2}^{2},tp_{2}^{3}\right)\right)=\left(\left(\frac{2\pi }{3},\frac{\pi }{6},\;\frac{\pi }{2}\right),\;\left(\frac{4\pi }{3},\;\frac{3\pi }{4},\;\frac{3\pi }{5}\right)\right)$. When performing rotation operations $R_{y}\left(TP_{1}\right)$ and $R_{y}\left(TP_{2}\right)$ on the two GHZ states, the resultant sequence $S_{initial}$ can be written as

(12) $$S_{\mathrm{initial}}=\left(\begin{array}{l} R_{y}\left(tp_{1}^{1}\right)⊗R_{y}\left(tp_{1}^{2}\right)⊗R_{y}\left(tp_{1}^{3}\right)|\varphi_{1}\rangle ,\\ R_{y}\left(tp_{2}^{1}\right)⊗R_{y}\left(tp_{2}^{1}\right)⊗R_{y}\left(tp_{2}^{1}\right)|\varphi_{6}\rangle \end{array}\right)=\left(\begin{array}{l} R_{y}\left(\frac{2\pi }{3}\right)⊗R_{y}\left(\frac{\pi }{6}\right)⊗R_{y}\left(\frac{\pi }{2}\right)|\varphi_{1}\rangle ,\\ R_{y}\left(\frac{4\pi }{3}\right)⊗R_{y}\left(\frac{3\pi }{4}\right)⊗R_{y}\left(\frac{3\pi }{5}\right)|\varphi_{6}\rangle \end{array}\right)$$

When receiving the sequence $S_{initial}$, Alice performs the rotation operations $R_{y}\left(\pi A_{1}\right)$ and $R_{y}\left(\pi A_{2}\right)$ on the two states in $S_{initial}$ to obtain a sequence $S_{A}$, which can be expressed as

(13) $$\begin{array}{l} S_{A}=\left(\begin{array}{l} \left(R_{y}\left(\pi x_{1}\right)⊗R_{y}\left(\pi x_{2}\right)⊗R_{y}\left(\pi x_{3}\right)\right)\left(R_{y}\left(tp_{1}^{1}\right)⊗R_{y}\left(tp_{1}^{2}\right)⊗R_{y}\left(tp_{1}^{3}\right)\right)|\varphi_{1}\rangle ,\\ \left(R_{y}\left(\pi x_{4}\right)⊗R_{y}\left(\pi x_{5}\right)⊗R_{y}\left(\pi x_{6}\right)\right)\left(R_{y}\left(tp_{2}^{1}\right)⊗R_{y}\left(tp_{2}^{1}\right)⊗R_{y}\left(tp_{2}^{1}\right)\right)|\varphi_{6}\rangle \end{array}\right)\\ =\left(\begin{array}{l} \left(R_{y}\left(0\right)⊗R_{y}\left(\pi \right)⊗R_{y}\left(0\right)\right)\left(R_{y}\left(\frac{2\pi }{3}\right)⊗R_{y}\left(\frac{\pi }{6}\right)⊗R_{y}\left(\frac{\pi }{2}\right)\right)|\varphi_{1}\rangle ,\\ \left(R_{y}\left(\pi \right)⊗R_{y}\left(0\right)⊗R_{y}\left(0\right)\right)\left(R_{y}\left(\frac{4\pi }{3}\right)⊗R_{y}\left(\frac{3\pi }{4}\right)⊗R_{y}\left(\frac{3\pi }{5}\right)\right)|\varphi_{6}\rangle \end{array}\right) \end{array}$$

We assume that the secret key $\Theta_{KA}=\left(KA_{1},KA_{2}\right)=\left(\left(ka_{1}^{1},ka_{1}^{2},ka_{1}^{3}\right),\left(ka_{2}^{1},ka_{2}^{2},ka_{2}^{3}\right)\right)=\left(\left(\frac{\pi }{3},\frac{5\pi }{6},\frac{\pi }{4}\right),\left(\frac{3\pi }{4},\frac{\pi }{2},\frac{\pi }{6}\right)\right)$. When performing rotation operations $R_{y}\left(KA_{1}\right)$ and $R_{y}\left(KA_{2}\right)$ on the two states in $S_{A}$, the resultant sequence $S_{Enc\_A}$ can be written as

(14) $$\begin{array}{l} S_{Enc\_A}=\left(\begin{array}{l} \left(R_{y}\left(ka_{1}^{1}\right)⊗R_{y}\left(ka_{1}^{2}\right)⊗R_{y}\left(ka_{1}^{3}\right)\right)\left(R_{y}\left(\pi x_{1}\right)⊗R_{y}\left(\pi x_{2}\right)⊗R_{y}\left(\pi x_{3}\right)\right)\left(R_{y}\left(tp_{1}^{1}\right)⊗R_{y}\left(tp_{1}^{2}\right)⊗R_{y}\left(tp_{1}^{3}\right)\right)|\varphi_{1}\rangle ,\\ \left(R_{y}\left(ka_{2}^{1}\right)⊗R_{y}\left(ka_{2}^{1}\right)⊗R_{y}\left(ka_{2}^{1}\right)\right)\left(R_{y}\left(\pi x_{4}\right)⊗R_{y}\left(\pi x_{5}\right)⊗R_{y}\left(\pi x_{6}\right)\right)\left(R_{y}\left(tp_{2}^{1}\right)⊗R_{y}\left(tp_{2}^{1}\right)⊗R_{y}\left(tp_{2}^{1}\right)\right)|\varphi_{6}\rangle \end{array}\right)\\ =\left(\begin{array}{l} \left(R_{y}\left(\frac{\pi }{3}\right)⊗R_{y}\left(\frac{5\pi }{6}\right)⊗R_{y}\left(\frac{\pi }{4}\right)\right)\left(R_{y}\left(0\right)⊗R_{y}\left(\pi \right)⊗R_{y}\left(0\right)\right)\left(R_{y}\left(\frac{2\pi }{3}\right)⊗R_{y}\left(\frac{\pi }{6}\right)⊗R_{y}\left(\frac{\pi }{2}\right)\right)|\varphi_{1}\rangle ,\\ \left(R_{y}\left(\frac{3\pi }{4}\right)⊗R_{y}\left(\frac{\pi }{2}\right)⊗R_{y}\left(\frac{\pi }{6}\right)\right)\left(R_{y}\left(\pi \right)⊗R_{y}\left(0\right)⊗R_{y}\left(0\right)\right)\left(R_{y}\left(\frac{4\pi }{3}\right)⊗R_{y}\left(\frac{3\pi }{4}\right)⊗R_{y}\left(\frac{3\pi }{5}\right)\right)|\varphi_{6}\rangle \end{array}\right) \end{array}$$

When receiving the secret key $\Theta_{KA}$ and obtaining the sequence $S_{Enc\_A}$, Bob performs rotation operations $R_{y}\left(-KA_{1}\right)$ and $R_{y}\left(-KA_{2}\right)$ on the two states in $S_{Enc\_A}$ to recover the sequence $S_{A}$. This process can be written as

(15) $$\begin{array}{l} S_{A}=\left(\begin{array}{l} \left(\begin{array}{l} \left(R_{y}\left(-ka_{1}^{1}\right)⊗R_{y}\left(-ka_{1}^{2}\right)⊗R_{y}\left(-ka_{1}^{3}\right)\right)\left(R_{y}\left(ka_{1}^{1}\right)⊗R_{y}\left(ka_{1}^{2}\right)⊗R_{y}\left(ka_{1}^{3}\right)\right)\\ \left(R_{y}\left(\pi x_{1}\right)⊗R_{y}\left(\pi x_{2}\right)⊗R_{y}\left(\pi x_{3}\right)\right)\left(R_{y}\left(tp_{1}^{1}\right)⊗R_{y}\left(tp_{1}^{2}\right)⊗R_{y}\left(tp_{1}^{3}\right)\right) \end{array}\right)|\varphi_{1}\rangle ,\\ \left(\begin{array}{l} \left(R_{y}\left(-ka_{2}^{1}\right)⊗R_{y}\left(-ka_{2}^{1}\right)⊗R_{y}\left(-ka_{2}^{1}\right)\right)\left(R_{y}\left(ka_{2}^{1}\right)⊗R_{y}\left(ka_{2}^{1}\right)⊗R_{y}\left(ka_{2}^{1}\right)\right)\\ \left(R_{y}\left(\pi x_{4}\right)⊗R_{y}\left(\pi x_{5}\right)⊗R_{y}\left(\pi x_{6}\right)\right)\left(R_{y}\left(tp_{2}^{1}\right)⊗R_{y}\left(tp_{2}^{1}\right)⊗R_{y}\left(tp_{2}^{1}\right)\right) \end{array}\right)|\varphi_{6}\rangle \end{array}\right)\\ =\left(\begin{array}{l} \left(R_{y}\left(\pi x_{1}\right)⊗R_{y}\left(\pi x_{2}\right)⊗R_{y}\left(\pi x_{3}\right)\right)\left(R_{y}\left(tp_{1}^{1}\right)⊗R_{y}\left(tp_{1}^{2}\right)⊗R_{y}\left(tp_{1}^{3}\right)\right)|\varphi_{1}\rangle ,\\ \left(R_{y}\left(\pi x_{4}\right)⊗R_{y}\left(\pi x_{5}\right)⊗R_{y}\left(\pi x_{6}\right)\right)\left(R_{y}\left(tp_{2}^{1}\right)⊗R_{y}\left(tp_{2}^{1}\right)⊗R_{y}\left(tp_{2}^{1}\right)\right)|\varphi_{6}\rangle \end{array}\right)\\ =\left(\begin{array}{l} \left(R_{y}\left(0\right)⊗R_{y}\left(\pi \right)⊗R_{y}\left(0\right)\right)\left(R_{y}\left(\frac{2\pi }{3}\right)⊗R_{y}\left(\frac{\pi }{6}\right)⊗R_{y}\left(\frac{\pi }{2}\right)\right)|\varphi_{1}\rangle ,\\ \left(R_{y}\left(\pi \right)⊗R_{y}\left(0\right)⊗R_{y}\left(0\right)\right)\left(R_{y}\left(\frac{4\pi }{3}\right)⊗R_{y}\left(\frac{3\pi }{4}\right)⊗R_{y}\left(\frac{3\pi }{5}\right)\right)|\varphi_{6}\rangle \end{array}\right) \end{array}$$

When performing rotation operations $R_{y}\left(\pi B_{1}\right)$ and $R_{y}\left(\pi B_{2}\right)$ on the two states in $S_{A}$, the resulting sequence $S_{B}$ can be written as

(16) $$\begin{array}{l} S_{B}=\left(\begin{array}{l} \left(R_{y}\left(\pi y_{1}\right)⊗R_{y}\left(\pi y_{2}\right)⊗R_{y}\left(\pi y_{3}\right)\right)\left(R_{y}\left(\pi x_{1}\right)⊗R_{y}\left(\pi x_{2}\right)⊗R_{y}\left(\pi x_{3}\right)\right)\left(R_{y}\left(tp_{1}^{1}\right)⊗R_{y}\left(tp_{1}^{2}\right)⊗R_{y}\left(tp_{1}^{3}\right)\right)|\varphi_{1}\rangle ,\\ \left(R_{y}\left(\pi y_{4}\right)⊗R_{y}\left(\pi y_{5}\right)⊗R_{y}\left(\pi y_{6}\right)\right)\left(R_{y}\left(\pi x_{4}\right)⊗R_{y}\left(\pi x_{5}\right)⊗R_{y}\left(\pi x_{6}\right)\right)\left(R_{y}\left(tp_{2}^{1}\right)⊗R_{y}\left(tp_{2}^{1}\right)⊗R_{y}\left(tp_{2}^{1}\right)\right)|\varphi_{6}\rangle \end{array}\right)\\ =\left(\begin{array}{l} \left(R_{y}\left(0\right)⊗R_{y}\left(\pi \right)⊗R_{y}\left(0\right)\right)\left(R_{y}\left(0\right)⊗R_{y}\left(\pi \right)⊗R_{y}\left(0\right)\right)\left(R_{y}\left(\frac{2\pi }{3}\right)⊗R_{y}\left(\frac{\pi }{6}\right)⊗R_{y}\left(\frac{\pi }{2}\right)\right)|\varphi_{1}\rangle ,\\ \left(R_{y}\left(0\right)⊗R_{y}\left(\pi \right)⊗R_{y}\left(0\right)\right)\left(R_{y}\left(\pi \right)⊗R_{y}\left(0\right)⊗R_{y}\left(0\right)\right)\left(R_{y}\left(\frac{4\pi }{3}\right)⊗R_{y}\left(\frac{3\pi }{4}\right)⊗R_{y}\left(\frac{3\pi }{5}\right)\right)|\varphi_{6}\rangle \end{array}\right) \end{array}$$

We assume that the secret key $\Theta_{KB}=\left(KB_{1},KB_{2}\right)=\left(\left(kb_{1}^{1},kb_{1}^{2},kb_{1}^{3}\right),\left(kb_{2}^{1},kb_{2}^{2},kb_{2}^{3}\right)\right)=\left(\left(\frac{\pi }{2},\frac{\pi }{3},\frac{5\pi }{6}\right),\left(\frac{\pi }{4},\frac{\pi }{8},\frac{\pi }{3}\right)\right)$. When performing rotation operations $R_{y}\left(KB_{1}\right)$ and $R_{y}\left(KB_{2}\right)$ on the two states in $S_{B}$, the resultant sequence $S_{Enc\_B}$ can be written as

(17) $$\begin{array}{l} S_{Enc\_B}=\left(\begin{array}{l} \left(\begin{array}{l} \left(R_{y}\left(kb_{1}^{1}\right)⊗R_{y}\left(kb_{1}^{2}\right)⊗R_{y}\left(kb_{1}^{3}\right)\right)\left(R_{y}\left(\pi y_{1}\right)⊗R_{y}\left(\pi y_{2}\right)⊗R_{y}\left(\pi y_{3}\right)\right)\\ \left(R_{y}\left(\pi x_{1}\right)⊗R_{y}\left(\pi x_{2}\right)⊗R_{y}\left(\pi x_{3}\right)\right)\left(R_{y}\left(tp_{1}^{1}\right)⊗R_{y}\left(tp_{1}^{2}\right)⊗R_{y}\left(tp_{1}^{3}\right)\right) \end{array}\right)|\varphi_{1}\rangle ,\\ \left(\begin{array}{l} \left(R_{y}\left(kb_{2}^{1}\right)⊗R_{y}\left(kb_{2}^{1}\right)⊗R_{y}\left(kb_{2}^{1}\right)\right)\left(R_{y}\left(\pi y_{4}\right)⊗R_{y}\left(\pi y_{5}\right)⊗R_{y}\left(\pi y_{6}\right)\right)\\ \left(R_{y}\left(\pi x_{4}\right)⊗R_{y}\left(\pi x_{5}\right)⊗R_{y}\left(\pi x_{6}\right)\right)\left(R_{y}\left(tp_{2}^{1}\right)⊗R_{y}\left(tp_{2}^{1}\right)⊗R_{y}\left(tp_{2}^{1}\right)\right) \end{array}\right)|\varphi_{6}\rangle \end{array}\right)\\ =\left(\begin{array}{l} \left(\begin{array}{l} \left(R_{y}\left(\frac{\pi }{2}\right)⊗R_{y}\left(\frac{\pi }{3}\right)⊗R_{y}\left(\frac{5\pi }{6}\right)\right)\left(R_{y}\left(0\right)⊗R_{y}\left(\pi \right)⊗R_{y}\left(0\right)\right)\\ \left(R_{y}\left(0\right)⊗R_{y}\left(\pi \right)⊗R_{y}\left(0\right)\right)\left(R_{y}\left(\frac{2\pi }{3}\right)⊗R_{y}\left(\frac{\pi }{6}\right)⊗R_{y}\left(\frac{\pi }{2}\right)\right) \end{array}\right)|\varphi_{1}\rangle ,\\ \left(\begin{array}{l} \left(R_{y}\left(\frac{\pi }{4}\right)⊗R_{y}\left(\frac{\pi }{8}\right)⊗R_{y}\left(\frac{\pi }{3}\right)\right)\left(R_{y}\left(0\right)⊗R_{y}\left(\pi \right)⊗R_{y}\left(0\right)\right)\\ \left(R_{y}\left(\pi \right)⊗R_{y}\left(0\right)⊗R_{y}\left(0\right)\right)\left(R_{y}\left(\frac{4\pi }{3}\right)⊗R_{y}\left(\frac{3\pi }{4}\right)⊗R_{y}\left(\frac{3\pi }{5}\right)\right) \end{array}\right)|\varphi_{6}\rangle \end{array}\right) \end{array}$$

When receiving the secret key $\Theta_{KB}$ and obtaining the sequence $S_{Enc\_B}$, the semi-honest TP performs rotation operations $R_{y}\left(-KB_{1}\right)$ and $R_{y}\left(-KB_{2}\right)$ on the two states in $S_{Enc\_B}$ to recover the sequence $S_{B}$. This process can be expressed as

(18) $$\begin{array}{l} S_{B}=\left(\begin{array}{l} \left(\begin{array}{l} \left(R_{y}\left(-kb_{1}^{1}\right)⊗R_{y}\left(-kb_{1}^{2}\right)⊗R_{y}\left(-kb_{1}^{3}\right)\right)\left(R_{y}\left(kb_{1}^{1}\right)⊗R_{y}\left(kb_{1}^{2}\right)⊗R_{y}\left(kb_{1}^{3}\right)\right)\left(R_{y}\left(\pi y_{1}\right)⊗R_{y}\left(\pi y_{2}\right)⊗R_{y}\left(\pi y_{3}\right)\right)\\ \left(R_{y}\left(\pi x_{1}\right)⊗R_{y}\left(\pi x_{2}\right)⊗R_{y}\left(\pi x_{3}\right)\right)\left(R_{y}\left(tp_{1}^{1}\right)⊗R_{y}\left(tp_{1}^{2}\right)⊗R_{y}\left(tp_{1}^{3}\right)\right) \end{array}\right)|\varphi_{1}\rangle ,\\ \left(\begin{array}{l} \left(R_{y}\left(-kb_{2}^{1}\right)⊗R_{y}\left(-kb_{2}^{1}\right)⊗R_{y}\left(-kb_{2}^{1}\right)\right)\left(R_{y}\left(kb_{2}^{1}\right)⊗R_{y}\left(kb_{2}^{1}\right)⊗R_{y}\left(kb_{2}^{1}\right)\right)\left(R_{y}\left(\pi y_{4}\right)⊗R_{y}\left(\pi y_{5}\right)⊗R_{y}\left(\pi y_{6}\right)\right)\\ \left(R_{y}\left(\pi x_{4}\right)⊗R_{y}\left(\pi x_{5}\right)⊗R_{y}\left(\pi x_{6}\right)\right)\left(R_{y}\left(tp_{2}^{1}\right)⊗R_{y}\left(tp_{2}^{1}\right)⊗R_{y}\left(tp_{2}^{1}\right)\right) \end{array}\right)|\varphi_{6}\rangle \end{array}\right)\\ =\left(\begin{array}{l} \left(\left(R_{y}\left(\pi y_{1}\right)⊗R_{y}\left(\pi y_{2}\right)⊗R_{y}\left(\pi y_{3}\right)\right)\left(R_{y}\left(\pi x_{1}\right)⊗R_{y}\left(\pi x_{2}\right)⊗R_{y}\left(\pi x_{3}\right)\right)\left(R_{y}\left(tp_{1}^{1}\right)⊗R_{y}\left(tp_{1}^{2}\right)⊗R_{y}\left(tp_{1}^{3}\right)\right)\right)|\varphi_{1}\rangle ,\\ \left(\left(R_{y}\left(\pi y_{4}\right)⊗R_{y}\left(\pi y_{5}\right)⊗R_{y}\left(\pi y_{6}\right)\right)\left(R_{y}\left(\pi x_{4}\right)⊗R_{y}\left(\pi x_{5}\right)⊗R_{y}\left(\pi x_{6}\right)\right)\left(R_{y}\left(tp_{2}^{1}\right)⊗R_{y}\left(tp_{2}^{1}\right)⊗R_{y}\left(tp_{2}^{1}\right)\right)\right)|\varphi_{6}\rangle \end{array}\right)\\ =\left(\begin{array}{l} \left(\left(R_{y}\left(0\right)⊗R_{y}\left(\pi \right)⊗R_{y}\left(0\right)\right)\left(R_{y}\left(0\right)⊗R_{y}\left(\pi \right)⊗R_{y}\left(0\right)\right)\left(R_{y}\left(\frac{2\pi }{3}\right)⊗R_{y}\left(\frac{\pi }{6}\right)⊗R_{y}\left(\frac{\pi }{2}\right)\right)\right)|\varphi_{1}\rangle ,\\ \left(\left(R_{y}\left(0\right)⊗R_{y}\left(\pi \right)⊗R_{y}\left(0\right)\right)\left(R_{y}\left(\pi \right)⊗R_{y}\left(0\right)⊗R_{y}\left(0\right)\right)\left(R_{y}\left(\frac{4\pi }{3}\right)⊗R_{y}\left(\frac{3\pi }{4}\right)⊗R_{y}\left(\frac{3\pi }{5}\right)\right)\right)|\varphi_{6}\rangle \end{array}\right) \end{array}$$

When performing rotation operations $R_{y}\left(-TP_{1}\right)$ and $R_{y}\left(-TP_{2}\right)$ on the two states in $S_{B}$, the resultant sequence $S_{final}$ can be given by

(19) $$\begin{array}{l} S_{final}=\left(\begin{array}{l} \left(\begin{array}{l} \left(R_{y}\left(-tp_{1}^{1}\right)⊗R_{y}\left(-tp_{1}^{2}\right)⊗R_{y}\left(-tp_{1}^{3}\right)\right)\left(R_{y}\left(\pi y_{1}\right)⊗R_{y}\left(\pi y_{2}\right)⊗R_{y}\left(\pi y_{3}\right)\right)\\ \left(R_{y}\left(\pi x_{1}\right)⊗R_{y}\left(\pi x_{2}\right)⊗R_{y}\left(\pi x_{3}\right)\right)\left(R_{y}\left(tp_{1}^{1}\right)⊗R_{y}\left(tp_{1}^{2}\right)⊗R_{y}\left(tp_{1}^{3}\right)\right) \end{array}\right)|\varphi_{1}\rangle ,\\ \left(\begin{array}{l} \left(R_{y}\left(-tp_{2}^{1}\right)⊗R_{y}\left(-tp_{2}^{1}\right)⊗R_{y}\left(-tp_{2}^{1}\right)\right)\left(R_{y}\left(\pi y_{4}\right)⊗R_{y}\left(\pi y_{5}\right)⊗R_{y}\left(\pi y_{6}\right)\right)\\ \left(R_{y}\left(\pi x_{4}\right)⊗R_{y}\left(\pi x_{5}\right)⊗R_{y}\left(\pi x_{6}\right)\right)\left(R_{y}\left(tp_{2}^{1}\right)⊗R_{y}\left(tp_{2}^{1}\right)⊗R_{y}\left(tp_{2}^{1}\right)\right) \end{array}\right)|\varphi_{6}\rangle \end{array}\right)\\ =\left(\begin{array}{l} \left(R_{y}\left(\pi y_{1}\right)⊗R_{y}\left(\pi y_{2}\right)⊗R_{y}\left(\pi y_{3}\right)\right)\left(R_{y}\left(\pi x_{1}\right)⊗R_{y}\left(\pi x_{2}\right)⊗R_{y}\left(\pi x_{3}\right)\right)|\varphi_{1}\rangle ,\\ \left(R_{y}\left(\pi y_{4}\right)⊗R_{y}\left(\pi y_{5}\right)⊗R_{y}\left(\pi y_{6}\right)\right)\left(R_{y}\left(\pi x_{4}\right)⊗R_{y}\left(\pi x_{5}\right)⊗R_{y}\left(\pi x_{6}\right)\right)|\varphi_{6}\rangle \end{array}\right)\\ =\left(\begin{array}{l} \left(R_{y}\left(0\right)⊗R_{y}\left(\pi \right)⊗R_{y}\left(0\right)\right)\left(R_{y}\left(0\right)⊗R_{y}\left(\pi \right)⊗R_{y}\left(0\right)\right)|\varphi_{1}\rangle ,\\ \left(R_{y}\left(0\right)⊗R_{y}\left(\pi \right)⊗R_{y}\left(0\right)\right)\left(R_{y}\left(\pi \right)⊗R_{y}\left(0\right)⊗R_{y}\left(0\right)\right)|\varphi_{6}\rangle \end{array}\right)\\ =\left(\begin{array}{l} \left(R_{y}\left(0\right)⊗R_{y}\left(0\right)⊗R_{y}\left(0\right)\right)|\varphi_{1}\rangle ,\\ \left(R_{y}\left(\pi \right)⊗R_{y}\left(\pi \right)⊗R_{y}\left(0\right)\right)|\varphi_{6}\rangle \end{array}\right)=\left(|\varphi_{1}\rangle ,|\varphi_{4}\rangle \right) \end{array}$$

When conducting GHZ-basis measurements on the two states in $S_{final}$, the measurement outcomes are $\left|\varphi_{1}\rangle \right.$ and $\left|\varphi_{4}\rangle \right.$. Since the measurement outcomes $\left|\varphi_{1}\rangle \right.$ and $\left|\varphi_{4}\rangle \right.$ are not consistent with the initially prepared GHZ states $\left|\varphi_{1}\rangle \right.$ and $\left|\varphi_{6}\rangle \right.$, TP can obtain the comparison result X≠Y.

**Figure 2:**
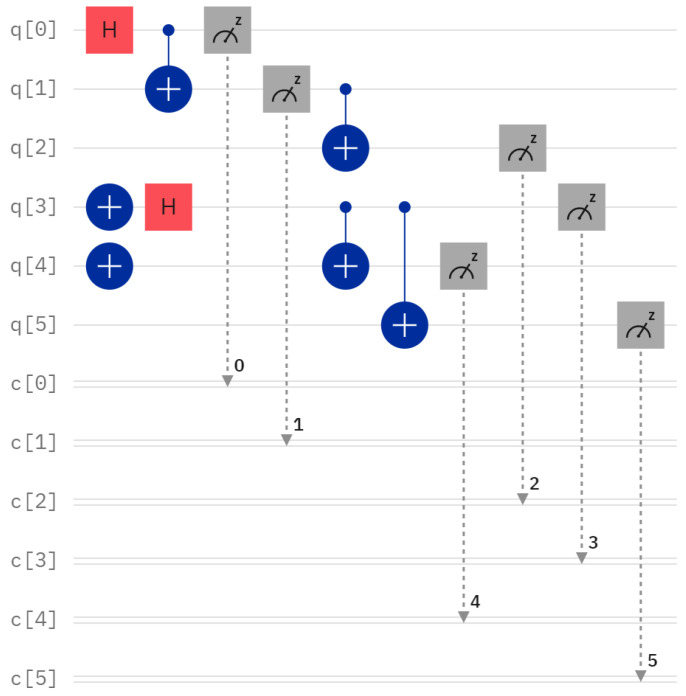
The quantum circuit of two GHZ states $\left|\varphi_{1}\right.$ and $\left|\varphi_{6}\rangle \right.$.

**Figure 3:**
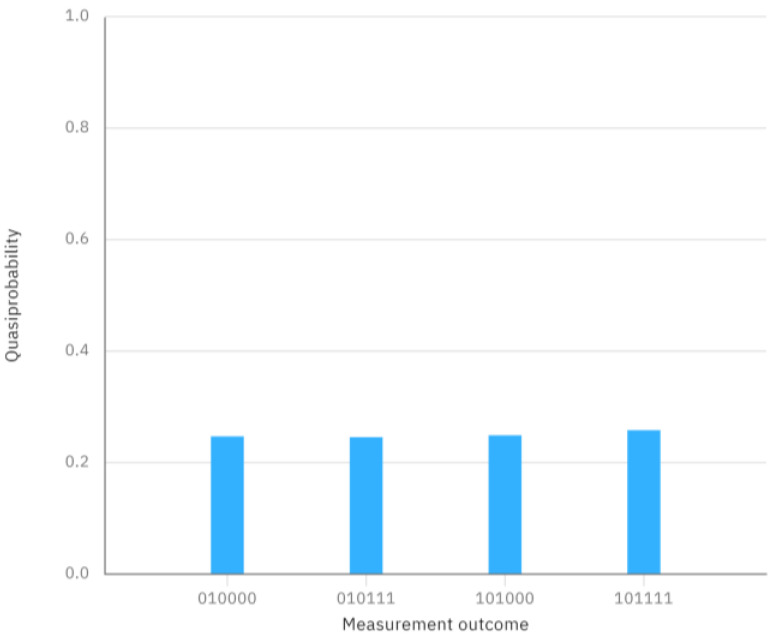
The measurement outcome in Figure 2.

**Figure 4:**
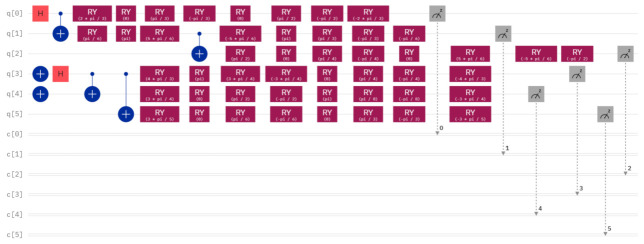
The quantum circuit corresponding to the concrete example.

**Figure 5:**
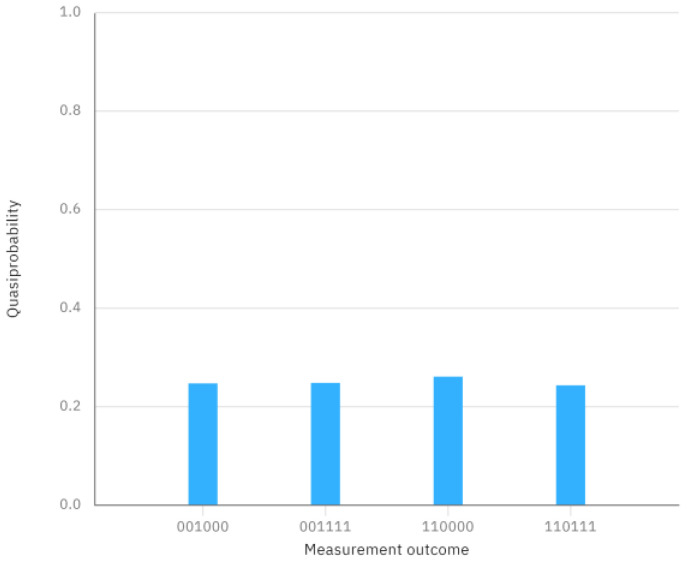
The measurement outcome in Figure 4.

For the concrete example mentioned above, the quantum circuit of two GHZ states $\left|\varphi_{1}\rangle \right.$ and $\left|\varphi_{6}\rangle \right.$, and its measurement outcome when executing this quantum circuit on IBM Quantum Composer are shown in Figure 2 and Figure 3, respectively. The quantum circuit corresponding to the concrete example and the final measurement outcome can be seen in Figure 4 and Figure 5, respectively.

From Figure 5, however, we can clearly observe that the measurement outcome when performing the quantum circuit of Figure 4 is different from the measurement outcome of the initially prepared two GHZ states in Figure 3. This discrepancy indicates that the measurement outcome is not consistent with the initially prepared GHZ states, suggesting that the comparison result is X≠Y.

## 5. Security Analysis

In this section, we will consider both external and participant attacks and demonstrate that our protocol is resistant to these attacks.

### 5.1. External Attacks

We assume that the external attacker, Eve, may adopt quantum attack methods (e.g., the intercept–measurement–resend attack, the entanglement-measure attack, and the Trojan-horse attacks) to steal the secrets of Alice or Bob. We will demonstrate that these attacks are ineffective due to the decoy-state method utilized in our protocol.

#### 5.1.1. The Intercept–Measurement–Resend Attack

**Figure 6:**
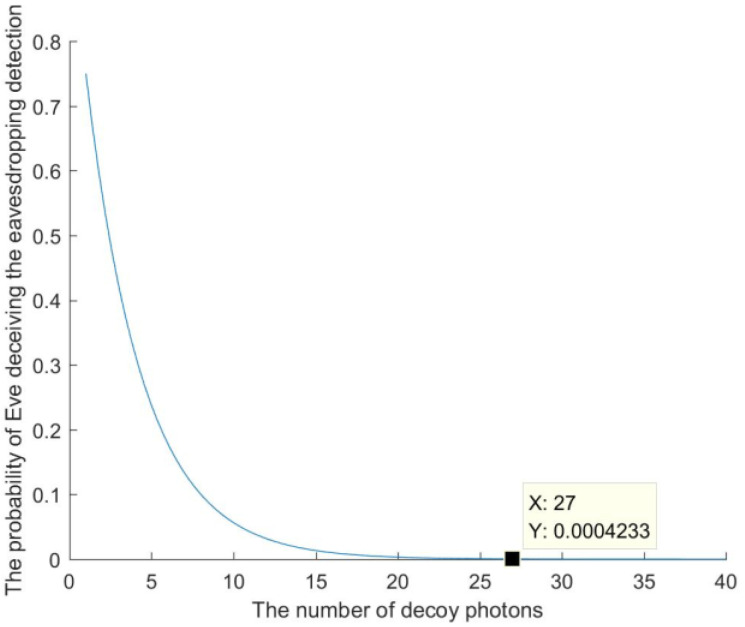
The relationship between the number of decoy photons and the probability that Eve will deceive the eavesdropping detection.

The intercept–measurement–resend attack occurs when an external attacker, Eve, intercepts the quantum sequence in the quantum channel, measures the intercepted quantum sequence to steal secrets of Alice or Bob, and then resends a fake quantum sequence to the receiver in place of the intercepted one. However, the attack will inevitably introduce errors due to eavesdropping detection between the quantum sequence sender and receiver. When the receiver receives the quantum sequence, she will measure the decoy states using the measurement basis announced by the sender and, then, send the measurement results back to the sender. Eve has no chance to know the specific state of the decoy states, resulting in inconsistencies between the intercepted decoy states and the measurement results. When Eve intercepts the sequence, there is a 50% probability of selecting the incorrect measurement basis, resulting in a correct and incorrect outcome of 50% each, respectively. For example, the sender prepares a decoy state with a quantum state $\left|1\right.\rangle$. The probability of Eve choosing the correct measurement basis with Z-basis ($\left\{\left|0\right.\rangle ,\left|1\right.\rangle \right\}$ basis) is 50%, and Eve will deceive the eavesdropping detection with a probability of 1. Simultaneously, the probability of Eve choosing the incorrect measurement basis with X-basis ($\left\{\left|+\right.\rangle ,\left|-\right.\rangle \right\}$ basis) is also 50%, and the probability of Eve deceiving the eavesdropping detection is 1/2. For *n* decoy states, the probability that Eve will deceive the eavesdropping detection is ${\left(3/4\right)}^{n}$. The relationship between the number of decoy photons and the probability of Eve deceiving the eavesdropping detection is shown in Figure 6. When the number of decoy photons, *n*, is large enough, the probability of Eve being discovered in the eavesdropping detection approaches 1 infinitely. Therefore, the intercept–measurement–resend attack launched by Eve is invalid for stealing the secrets of Alice or Bob.

#### 5.1.2. The Entanglement-Measure Attack

The entanglement-measure attack occurs when Eve intercepts the quantum particles during transmission and, then, utilizes unitary operations to entangle her auxiliary particles $\left|\varepsilon \right.\rangle$ with the intercepted particles. She then measures the auxiliary particles to obtain private information about Alice or Bob. 

When using the unitary operation U to entangle the intercepted particle with quantum states $\left|0\right.\rangle$ and $\left|1\right.\rangle$, this process can be expressed as

(20) $$U{|0,\varepsilon \rangle }_{TE}=\alpha |0\rangle |\varepsilon_{00}\rangle +\beta |1\rangle |\varepsilon_{01}\rangle$$

(21) $$U{|1,\varepsilon \rangle }_{TE}=\gamma |0\rangle |\varepsilon_{10}\rangle +\delta |1\rangle |\varepsilon_{11}\rangle$$

where the subscript T and E denote the intercepted particle and the auxiliary particle, respectively. Four states $\left|\varepsilon_{00}\right.\rangle ,\left|\varepsilon_{01}\right.\rangle ,\left|\varepsilon_{10}\right.\rangle ,\;and\;\left|\varepsilon_{11}\right.\rangle$ are pure states determined by the unitary operation U. The parameters α,β,γ,δ should meet the specified conditions: $\alpha^{2}+\beta^{2}=1$, $\gamma^{2}+\delta^{2}=1$.

When using the unitary operation U to entangle the intercepted particles with quantum state $\left|+\right.\rangle$ and $\left|-\right.\rangle$, this process can be expressed as

(22) $$\begin{array}{l} U{|+,\varepsilon \rangle }_{TE}=\frac{1}{\sqrt{2}}\left(\alpha |0\rangle |\varepsilon_{00}\rangle +\beta |\varepsilon_{01}\rangle |1\rangle +\gamma |0\rangle |\varepsilon_{10}\rangle +\delta |1\rangle |\varepsilon_{11}\rangle \right)\\ =\frac{1}{2}\left(\begin{array}{l} |+\rangle \left(\alpha |\varepsilon_{00}\rangle +\beta |\varepsilon_{01}\rangle +\gamma |\varepsilon_{10}\rangle +\delta |e_{11}\rangle \right)\\ +|-\rangle \left(\alpha |\varepsilon_{00}\rangle -\beta |\varepsilon_{01}\rangle +\gamma |\varepsilon_{10}\rangle -\delta |e_{11}\rangle \right) \end{array}\right) \end{array}$$

(23) $$\begin{array}{l} U{|-,\varepsilon \rangle }_{TE}=\frac{1}{\sqrt{2}}\left(\alpha |0\rangle |\varepsilon_{00}\rangle +\beta |\varepsilon_{01}\rangle |1\rangle -\gamma |0\rangle |\varepsilon_{10}\rangle -\delta |1\rangle |\varepsilon_{11}\rangle \right)\\ =\frac{1}{2}\left(\begin{array}{l} |+\rangle \left(\alpha |\varepsilon_{00}\rangle +\beta |\varepsilon_{01}\rangle -\gamma |\varepsilon_{10}\rangle -\delta |e_{11}\rangle \right)\\ +|-\rangle \left(\alpha |\varepsilon_{00}\rangle -\beta |\varepsilon_{01}\rangle -\gamma |\varepsilon_{10}\rangle +\delta |e_{11}\rangle \right) \end{array}\right) \end{array}$$

In our protocol, the eavesdropping detection process occurs throughout the entire quantum sequence transmission. If the decoy state stays in $\left\{\left|0\right.\rangle ,\left|1\right.\rangle \right\}$ basis and Eve tries to trick the eavesdropping detection, the parameters in Equations (20) and (21) should be set as β=γ=0. If the decoy state stays in $\left\{\left|+\right.\rangle ,\left|-\right.\rangle \right\}$ basis and Eve tries to trick the eavesdropping detection, $\alpha \left|\varepsilon_{00}\right.\rangle -\beta \left|\varepsilon_{01}\right.\rangle +\gamma \left|\varepsilon_{10}\right.\rangle -\delta \left|e_{11}\right.\rangle$ and $\alpha \left|\varepsilon_{00}\right.\rangle +\beta \left|\varepsilon_{01}\right.\rangle -\gamma \left|\varepsilon_{10}\right.\rangle -\delta \left|e_{11}\right.\rangle$ should be a zero vector. Thus, we can conclude that $\alpha \left|\varepsilon_{00}\right.\rangle =\delta \left|e_{11}\right.\rangle$. Finally, Equations (20)–(23) can be reformulated as

(24) $$U{|0,\varepsilon \rangle }_{TE}=\alpha |0\rangle |\varepsilon_{00}\rangle$$

(25) $$U{|1,\varepsilon \rangle }_{TE}=\delta |1\rangle |\varepsilon_{11}\rangle =\alpha |1\rangle |\varepsilon_{00}\rangle$$

(26) $$\begin{array}{l} U{|+,\varepsilon \rangle }_{TE}=\frac{1}{2}|+\rangle \left(\alpha |\varepsilon_{00}\rangle +\beta |\varepsilon_{01}\rangle +\gamma |\varepsilon_{10}\rangle +\delta |e_{11}\rangle \right)\\ =\frac{1}{2}|+\rangle \left(\alpha |\varepsilon_{00}\rangle +0+0+\delta |e_{11}\rangle \right)=\alpha |+\rangle |\varepsilon_{00}\rangle \end{array}$$

(27) $$\begin{array}{l} U{|-,\varepsilon \rangle }_{TE}=\frac{1}{2}|-\rangle \left(\alpha |\varepsilon_{00}\rangle -\beta |\varepsilon_{01}\rangle -\gamma |\varepsilon_{10}\rangle +\delta |e_{11}\rangle \right)\\ =\frac{1}{2}|-\rangle \left(\alpha |\varepsilon_{00}\rangle -0-0+\delta |e_{11}\rangle \right)=\alpha |-\rangle |\varepsilon_{00}\rangle \end{array}$$

According to Equations (24)–(27), it can be inferred that the tensor product of the intercepted particle and the auxiliary particle results in a product of two quantum states, indicating that the auxiliary particle is independent of the intercepted particle. Even if Eve measures the auxiliary particles, she cannot obtain any information about the intercepted particles.

Additionally, assuming that Eve entangles her auxiliary particles with the transmitted GHZ states, her behavior will not succeed since the transmitted GHZ states are encrypted by the rotation operations, which are unknown to her. Therefore, the rotation operations ensure the concealment of the transmitted quantum states from external attackers, and the decoy state method can be employed to safeguard the security of the quantum channel. 

#### 5.1.3. The Trojan-Horse Attacks

The Trojan-horse attacks [34], including the delay-photon Trojan-horse attack and the invisible photon eavesdropping Trojan-horse attack, mainly occur in two-way quantum communication. Since the quantum states in our protocol are transmitted in a circular mode, there may be potential security risks due to Trojan-horse attacks. However, these attacks can be detected using existing techniques. The Wavelength Quantum Filter (WQF) can be used to remove invisible photons using optical filters, and the Photons Number Splitter (PNS) can be used to separate legitimate photons from delayed photons. Once these attacks are detected, the protocol will be aborted and restarted.

### 5.2. Participant Attacks

The participants who have access to immediate results may deduce private information by launching more powerful attacks, which poses a security challenge for our protocol [35]. In the following, three cases of attacks will be analyzed in detail. 

#### 5.2.1. TP’s Attack

In the proposed protocol, TP is assumed to be semi-honest, which means she cannot conspire with Alice and Bob but may attempt to steal the secrets of Alice and Bob. If TP wants to learn information about Alice or Bob’s inputs, she can act as an external attacker and perform the corresponding attacks. However, her behavior will be detected during the eavesdropping detection process as discussed in Section 5.1. In this scenario, we are examining a special case where TP executes an intercept–resend attack on the sequence sent from Alice to Bob. TP intercepts the sequence $S_{Enc\_A}^{\prime }$ and resends a fake sequence to Bob. When Alice announces the positions where decoy states were inserted, TP discards the decoy states in $S_{Enc\_A}^{\prime }$ and obtains the sequence $S_{Enc\_A}$ containing Alice’s encoding inputs. Although this attack will be detected, TP may perform rotation operations $R_{y}\left(-TP_{j}\right)$ on the *j*-th GHZ states in $S_{Enc\_A}$ to obtain a sequence $S_{A\to B}$ and, then, conduct GHZ-basis measurements on the *j*-th states in $S_{A\to B}$ to obtain the measurement outcomes. TP may deduce Alice’s private inputs by comparing the measurement outcomes with the initially prepared GHZ states. Unfortunately, the sequence $S_{Enc\_A}$ is obtained by performing rotation operations $R_{y}\left(KA_{j}\right)$ on the *j*-th states in $S_{A}$, and the secret key $\Theta_{KA}$ will be disclosed by Alice under the condition that no external eavesdropper exists. TP has no chance to obtain $\Theta_{KA}$, and she cannot obtain the sequence $S_{A}$, making it inaccessible to deduce the rotation operations $R_{y}\left(KA_{j}\right)$. TP’s lack of knowledge about $R_{y}\left(KA_{j}\right)$ is equivalent to her inability to access Alice’s private inputs. Additionally, TP can leverage the benefits of preparing the initial GHZ states to compute the comparison results and infer the inputs of Alice or Bob based on the final measurement outcomes. In this case, the inputs of Alice and Bob will not be disclosed since each measurement outcome only reveals the XOR value of three bits. Therefore, our protocol is secure against TP’s attacks. 

#### 5.2.2. Alice’s Attack

For Alice, she may send a fake sequence $S_{A}$ to Bob, intercept the sequence $S_{Enc\_B}^{\prime }$ sent from Bob to TP, and then, resend another fake sequence to TP. Once the secret key $\Theta_{KB}$ is announced by Bob, Alice can recover $S_{B}$ by performing rotation operation $R_{y}\left(-KB_{j}\right)$ on the *j*-th states in $S_{Enc\_B}$. However, this attack method is invalid in our protocol. Once Alice intercepts the sequence $S_{Enc\_B}$, her behavior will inevitably be detected during the eavesdropping between Bob and TP. Once the eavesdropper intervenes in the transmission process, Bob will not disclose the secret key to TP. The protocol will be aborted and restarted. Therefore, Alice has no chance of learning Bob’s inputs.

#### 5.2.3. Bob’s Attack

For Bob, he can measure the sequence $S_{B}$ in the GHZ-basis and obtain the measurement outcomes. He may deduce which rotation operations have been performed by Alice by comparing the measurement outcomes with the initially prepared GHZ states and learn Alice’s inputs. However, this method does not work. On the one hand, the initially prepared GHZ states are only known to TP who cannot conspire with any participants, resulting in Bob having no chance to know them. On the other hand, Bob may attempt to obtain the sequence $S_{initial}$ by performing an intercept–resend attack on the sequence $S_{initial}^{\prime }$ and infer the initially prepared GHZ states. Although his behavior will be detected, and he can obtain $S_{initial}$, he still has no chance to learn the initially prepared GHZ states. This is because the sequence $S_{initial}$ is obtained by performing rotation operations $R_{y}\left(TP_{j}\right)$ on the *j*-th GHZ states, and no one can know the initially prepared GHZ states without knowing the secret key $\Theta_{TP}$. Without knowledge of the initially prepared GHZ states, Bob is unable to acquire information about Alice’s inputs.

## 6. Efficiency Analysis and Comparison

### 6.1. Efficiency Analysis

The qubit efficiency [36], as a measure of the utilization rate of quantum states, can be defined as

(28) $$\eta_{e}=\frac{\eta_{c}}{\eta_{t}}$$

where $\eta_{e}$ denotes the qubit efficiency of the QPC protocol, $\eta_{c}$ represents the number of compared classical bits, and $\eta_{t}$ denotes the total consumed qubits excluding the decoy photons used. In our protocol, one GHZ state can be compared to three bits of classical information in each comparison, and we can obtain $\eta_{c}=\eta_{t}$. Therefore, the qubit efficiency of our protocol is 100%.

### 6.2. Comparison

We compare our protocol with QPC protocols proposed in Refs. [9,16,18,22,26] in Table 2. The comparison between our protocol and other QPC protocols based on GHZ state is shown in Table 3.

Compared with QPC protocols in Refs. [9,16,18,22,26,37,38,39,40], our protocol has the following advantages. First, our protocol does not require QKD protocol for sharing a secret key to ensure the security of the inputs. This results in no consumption of quantum resources for key sharing, unlike QKD-based QPC protocols [8,16,18,22,37,38,40]. Secondly, the quantum sequence is transmitted between the TP and the two users in a circular mode. The inputs of the two users are encoded into the transmitted quantum sequence, leading to the multiplexing of quantum resources and improving the utilization of these resources. Third, our protocol reaches the maximum theoretical efficiency of 100%, because one GHZ state can be compared to three bits of classical information in each comparison. To sum up, our protocol requires no quantum resources for sharing a secret key, and it has shown improved performance in qubit efficiency and the utilization of quantum resources.

## 7. Conclusions

In this article, we propose an efficient QPC protocol based on GHZ states. With the assistance of a semi-honest TP, two users can compare their secrets by utilizing the properties of GHZ states and rotation operations. Compared with other QPC protocols, one of the advantages of our protocol is that it utilizes secret keys distributed through classical channels instead of QKD protocols to share a secret key, which results in no consumption of quantum resources for key sharing. The quantum sequence is transmitted between the TP and two users in a circular mode. The inputs of the two users are encoded into the transmitted quantum sequence, leading to the multiplexing of quantum resources and improving the utilization of quantum resources. More importantly, our protocol achieves a qubit efficiency of 100%, which is the theoretical maximum.

## Figures and Tables

**Table 1:** The resultant states without considering the global phase.

	$|\varphi_{1}\rangle$	$|\varphi_{2}\rangle$	$|\varphi_{3}\rangle$	$|\varphi_{4}\rangle$	$|\varphi_{5}\rangle$	$|\varphi_{6}\rangle$	$|\varphi_{7}\rangle$	$|\varphi_{8}\rangle$
$\theta_{1}\theta_{2}\theta_{3}=000$	$|\varphi_{1}\rangle$	$|\varphi_{2}\rangle$	$|\varphi_{3}\rangle$	$|\varphi_{4}\rangle$	$|\varphi_{5}\rangle$	$|\varphi_{6}\rangle$	$|\varphi_{7}\rangle$	$|\varphi_{8}\rangle$
$\theta_{1}\theta_{2}\theta_{3}=00\pi$	$|\varphi_{8}\rangle$	$|\varphi_{7}\rangle$	$|\varphi_{6}\rangle$	$|\varphi_{5}\rangle$	$|\varphi_{4}\rangle$	$|\varphi_{3}\rangle$	$|\varphi_{2}\rangle$	$|\varphi_{1}\rangle$
$\theta_{1}\theta_{2}\theta_{3}=0\pi 0$	$|\varphi_{6}\rangle$	$|\varphi_{5}\rangle$	$|\varphi_{8}\rangle$	$|\varphi_{7}\rangle$	$|\varphi_{2}\rangle$	$|\varphi_{1}\rangle$	$|\varphi_{4}\rangle$	$|\varphi_{3}\rangle$
$\theta_{1}\theta_{2}\theta_{3}=0\pi \pi$	$|\varphi_{3}\rangle$	$|\varphi_{4}\rangle$	$|\varphi_{1}\rangle$	$|\varphi_{2}\rangle$	$|\varphi_{7}\rangle$	$|\varphi_{8}\rangle$	$|\varphi_{5}\rangle$	$|\varphi_{6}\rangle$
$\theta_{1}\theta_{2}\theta_{3}=\pi 00$	$|\varphi_{4}\rangle$	$|\varphi_{3}\rangle$	$|\varphi_{2}\rangle$	$|\varphi_{1}\rangle$	$|\varphi_{8}\rangle$	$|\varphi_{7}\rangle$	$|\varphi_{6}\rangle$	$|\varphi_{5}\rangle$
$\theta_{1}\theta_{2}\theta_{3}=\pi 0\pi$	$|\varphi_{5}\rangle$	$|\varphi_{6}\rangle$	$|\varphi_{7}\rangle$	$|\varphi_{8}\rangle$	$|\varphi_{1}\rangle$	$|\varphi_{2}\rangle$	$|\varphi_{3}\rangle$	$|\varphi_{4}\rangle$
$\theta_{1}\theta_{2}\theta_{3}=\pi \pi 0$	$|\varphi_{7}\rangle$	$|\varphi_{8}\rangle$	$|\varphi_{5}\rangle$	$|\varphi_{6}\rangle$	$|\varphi_{3}\rangle$	$|\varphi_{4}\rangle$	$|\varphi_{1}\rangle$	$|\varphi_{2}\rangle$
$\theta_{1}\theta_{2}\theta_{3}=\pi \pi \pi$	$|\varphi_{2}\rangle$	$|\varphi_{1}\rangle$	$|\varphi_{4}\rangle$	$|\varphi_{3}\rangle$	$|\varphi_{6}\rangle$	$|\varphi_{5}\rangle$	$|\varphi_{8}\rangle$	$|\varphi_{7}\rangle$

**Table 2:** The comparison between our protocol and some previous protocols.

	Ref. [9]	Ref. [16]	Ref. [18]	Ref. [22]	Ref. [26]	Ours
Quantum resource	Single photons	Bell states	Eight-qubit entangled state	Four-qubit cluster state and extended Bell state	Five-particle cluster state	GHZ states
Unitary operation	No	No	No	No	Yes	Yes
Entanglement swapping	No	Yes	No	Yes	No	No
QKD method	Yes	Yes	Yes	Yes	No	No
Quantum measurement	Single-particle	GHZ-basis	single-particle	Bell-basis and extend Bell basis	single-particle	GHZ-basis
Qubit efficiency	33%	50%	25%	50%	40%	100%

**Table 3:** The comparison between our protocol and other QPC protocols based on GHZ state.

	Ref. [37]	Ref. [38]	Ref. [39]	Ref. [40]	Ours
Quantum resource	Hyperentangled GHZ state	4D GHZ-like states	GHZ state	four-particle GHZ state	GHZ states
Unitary operation	No	No	No	Yes	Yes
Entanglement swapping	Yes	No	Yes	No	No
QKD method	Yes	Yes	No	Yes	No
Quantum measurement	Bell-basis	single-particle	Bell-basis	Bell-basis and single-particle	GHZ-basis
Qubit efficiency	66%	33%	33%	75%	100%

## Data Availability

Data is contained within the article.

## References

[1] Shor P.W. (1999). Polynomial-time algorithms for prime factorization and discrete logarithms on a quantum computer. SIAM Rev..

[2] Grover L.K. (1997). Quantum mechanics helps in searching for a needle in a haystack. Phys. Rev. Lett..

[3] Yang Y.G., Wen Q.Y. (2009). An efficient two-party quantum private comparison protocol with decoy photons and two-photon entanglement. J. Phys. A Math. Theor..

[4] Chen X.B., Xu G., Niu X.X., Wen Q.Y., Yang Y.X. (2010). An efficient protocol for the private comparison of equal information based on the triplet entangled state and single-particle measurement. Opt. Commun..

[5] Lin J., Tseng H.Y., Hwang T. (2011). Intercept–resend attacks on Chen et al.’s quantum private comparison protocol and the improvements. Opt. Commun..

[6] Tseng H.Y., Lin J., Hwang T. (2012). New quantum private comparison protocol using EPR pairs. Quantum Inf. Process..

[7] Jia H.Y., Wen Q.Y., Li Y.B., Gao F. (2012). Quantum private comparison using genuine four-particle entangled states. Int. J. Theor. Phys..

[8] Huang W., Wen Q.Y., Liu B., Gao F., Sun Y. (2013). Robust and efficient quantum private comparison of equality with collective detection over collective-noise channels. Sci. China Phys. Mech. Astron..

[9] Sun Z., Yu J., Wang P., Xu L., Wu C. (2015). Quantum private comparison with a malicious third party. Quantum Inf. Process..

[10] Kou T.Y., Che B.C., Dou Z., Chen X.-B., Lai Y.-P., Li J. (2022). Efficient quantum private comparison protocol utilizing single photons and rotational encryption. Chin. Phys. B.

[11] Huang X., Zhang W.F., Zhang S.B. (2023). Efficient multiparty quantum private comparison protocol based on single photons and rotation encryption. Quantum Inf. Process..

[12] Ye T.Y., Ji Z.X. (2017). Two-party quantum private comparison with five-qubit entangled states. Int. J. Theor. Phys..

[13] Li J., Wang Z., Yang J., Ye C., Che F. (2023). A Semi-Quantum Private Comparison Base on W-States. Entropy.

[14] Ji Z.X., Zhang H.G., Fan P.R. (2019). Two-party quantum private comparison protocol with maximally entangled seven-qubit state. Mod. Phys. Lett. A.

[15] Ji Z., Zhang H., Wang H. (2019). Quantum private comparison protocols with a number of multi-particle entangled states. IEEE Access.

[16] Huang X., Zhang S.B., Chang Y., Hou M., Cheng W. (2021). Efficient quantum private comparison based on entanglement swapping of bell states. Int. J. Theor. Phys..

[17] Wu W., Wu J., Guo L. (2023). Multi-Party Quantum Private Comparison Based on Bell States. Entropy.

[18] Fan P., Rahman A.U., Ji Z., Ji X., Hao Z., Zhang H. (2022). Two-party quantum private comparison based on eight-qubit entangled state. Mod. Phys. Lett. A.

[19] Hong-Ming P. (2017). Quantum private comparison based on χ-type entangled states. Int. J. Theor. Phys..

[20] Ji Z.X., Ye T.Y. (2016). Quantum private comparison of equal information based on highly entangled six-qubit genuine state. Commun. Theor. Phys..

[21] Li J., Che F., Wang Z., Fu A. (2023). Efficient Quantum Private Comparison without Sharing a Key. Entropy.

[22] Li C., Chen X., Li H., Yang Y., Li J. (2019). Efficient quantum private comparison protocol based on the entanglement swapping between four-qubit cluster state and extended Bell state. Quantum Inf. Process..

[23] Sun Z., Long D. (2013). Quantum private comparison protocol based on cluster states. Int. J. Theor. Phys..

[24] Zhou M.K. (2018). Improvements of quantum private comparison protocol based on cluster states. Int. J. Theor. Phys..

[25] Zha X.W., Yu X.Y., Cao Y., Wang S.-K. (2018). Quantum private comparison protocol with five-particle cluster states. Int. J. Theor. Phys..

[26] Chang Y., Zhang W.B., Zhang S.B., Wang H.-C., Yan L.-L., Han G.-H., Sheng Z.-W., Huang Y.-Y., Suo W., Xiong J.-X. (2016). Quantum private comparison of equality based on five-particle cluster state. Commun. Theor. Phys..

[27] Ye T.Y. (2017). Quantum private comparison via cavity QED. Commun. Theor. Phys..

[28] Lang Y.F. (2020). Quantum gate-based quantum private comparison. Int. J. Theor. Phys..

[29] Zhang J.W., Xu G., Chen X.B., Chang Y., Dong Z.-C. (2023). Improved multiparty quantum private comparison based on quantum homomorphic encryption. Phys. A Stat. Mech. Its Appl..

[30] Huang X., Chang Y., Cheng W., Hou M., Zhang S.B. (2022). Quantum private comparison of arbitrary single qubit states based on swap test. Chin. Phys. B.

[31] Wang N., Zhang X., Zhang X., Lin S. (2022). (t, n) Threshold Quantum Secret Sharing Using Rotation Operation. Int. J. Theor. Phys..

[32] Kang M.S., Hong C.H., Heo J., Lim J.-I., Yang H.-J. (2015). Quantum signature scheme using a single qubit rotation operator. Int. J. Theor. Phys..

[33] Sun Z., Huang J., Wang P. (2016). Efficient multiparty quantum key agreement protocol based on commutative encryption. Quantum Inf. Process..

[34] Jain N., Anisimova E., Khan I., Makarov V., Marquardt C., Leuchs G. (2014). Trojan-horse attacks threaten the security of practical quantum cryptography. New J. Phys..

[35] Huang X., Zhang W., Zhang S. (2024). Practical quantum protocols for blind millionaires’ problem based on rotation encryption and swap test. Phys. A Stat. Mech. Appl..

[36] Cabello A. (2000). Quantum key distribution in the Holevo limit. Phys. Rev. Lett..

[37] Gianni J., Qu Z. (2021). New quantum private comparison using hyperentangled ghz state. J. Quantum Comput..

[38] Liu C., Zhou S., Gong L.H., Chen H.-Y. (2023). Quantum private comparison protocol based on 4D GHZ-like states. Quantum Inf. Process..

[39] Liu W., Wang Y.B. (2012). Quantum private comparison based on GHZ entangled states. Int. J. Theor. Phys..

[40] Xu Q.D., Chen H.Y., Gong L.H., Zhou N.R. (2020). Quantum private comparison protocol based on four-particle GHZ states. Int. J. Theor. Phys..

